# Supplementary material for: Content-rich biological network constructed by mining PubMed abstracts
Source: BMC Bioinformatics. 2004 Oct 8;5:147. doi: 10.1186/1471-2105-5-147 (PMC528731; doi:10.1186/1471-2105-5-147)
Supplement: Additional File 2 — The original results of the above study (non-essential files are deleted to keep the file size under the limit set by BMC bioinformatics). [file 1471-2105-5-147-S2.bz2 › chilibotAdditionalFile2/dip20/163ID8612709E569/html/BMPR1B_ACVR2.html]

 


 **BMPR1B** and **ACVR2** 
  
Found 15 abstracts in PubMed, retrieved 15.  
 

 What does Google say? 
 PDF only 
| .edu only 

---

**Interactive relationship** (e.g. stimulation, inhibition, etc)

**Neutral relationship**- BMP 4 and GDF 5 are known to bind to activin receptor  [ **ACVR2** ]  like kinase 3 ALK 3 and or ALK 6  [ **BMPR1B** ]  also termed BMP type IA and type IB receptors, respectively.  Ref: 11282024 J Cell Sci, 2001

**Non-interactive relationship** (e.g. studied together, co-existance, homology, etc.)

- We then investigated whether the sensitivity of the early long bone rudiments to OP 1 correlated with high expression of the OP 1 binding type I serine threonine kinase receptors activin receptor  [ **ACVR2** ]  like kinase ALK 2 ActR I, ALK 3 BMPR IA or ALK 6  [ **BMPR1B** ]  BMPR IB at this early stage.  Ref: 10705576 Growth Factors, 2000
- Moreover, GDF 5 transduced the signal efficiently by ALK 6  [ **BMPR1B** ]  BMPR IB in the presence of BMPR II or ActR II  [ **ACVR2** ] .  Ref: 9125848 Kokubyo Gakkai Zasshi, 1997
- BMP type II receptor BMPR II is a type II serine threonine kinase receptor, which transduces signals for BMPs through heteromeric complexes with type I receptors, including activin receptor  [ **ACVR2** ]  like kinase 2 ALK2, ALK3 BMPR IA, and ALK6  [ **BMPR1B** ]  BMPR IB.  Ref: 10772805 Dev Biol, 2000
- a series of related receptors, denoted activin receptor  [ **ACVR2** ]  like kinase ALK 1 to 5, have recently been identified, and ALK 6  [ **BMPR1B** ]  is described here.  Ref: 8140412 Science, 1994
- A mechanistic explanation for increased pSmad1 5 staining during inflammation was provided by the upregulated expression of all the BMP type I receptors, i.e., activin receptor  [ **ACVR2** ]  like kinase ALK 2, ALK3, and ALK6  [ **BMPR1B** ] , in the inflamed bronchial epithelial cells.  Ref: 12151307 Am J Respir Cell Mol Biol, 2002
- Activin receptor  [ **ACVR2** ]  like kinase ALK 1, ALK 2, ALK 3, and ALK 6  [ **BMPR1B** ]  were able to reproduce the hypertrophic maturation of chondrocytes induced by BMP 2.  Ref: 12082094 J Biol Chem, 2002
- To identify the receptors for BMP 15, we utilized recombinant extracellular domains of individual transforming growth factor beta superfamily receptors and found that activin receptor  [ **ACVR2** ]  like kinase 6  [ **BMPR1B** ]  extracellular domain most effectively co immunoprecipitates with BMP 15.  Ref: 12419820 J Biol Chem, 2003
